# Influence of TiO_2_ Nanoparticles on Liquid Crystalline, Structural and Electrochemical Properties of (8Z)-N-(4-((Z)-(4-pentylphenylimino)methyl)benzylidene)-4-pentylbenzenamine

**DOI:** 10.3390/ma12071097

**Published:** 2019-04-02

**Authors:** Anna Różycka, Krzysztof Artur Bogdanowicz, Natalia Górska, Jakub Rysz, Monika Marzec, Agnieszka Iwan, Robert Pich, Adam Januszko

**Affiliations:** 1 Institute of Physics, Jagiellonian University, S. Lojasiewicza 11, 30-348 Krakow, Poland; a.rozycka@doctoral.uj.edu.pl (A.R.); jakub.rysz@uj.edu.pl (J.R.); 2 Military Institute of Engineer Technology, Obornicka 136, 50-961 Wroclaw, Poland; bogdanowicz@witi.wroc.pl; 3 Faculty of Chemistry, Jagiellonian University, Gronostajowa 2, 30-387 Kraków, Poland; gorska@chemia.uj.edu.pl; 4 General Tadeusz Kosciuszko Military University of Land Forces, Wroclaw, MULF Wroclaw, Faculty of Security Studies, Czajkowskiego 109, 51-147 Wroclaw, Poland; robert.pich@awl.edu.pl (R.P.); adam.januszko@awl.edu.pl (A.J.)

**Keywords:** TiO_2_, imines, azomethines, hybrids, FT-IR, thermographic camera, organic devices

## Abstract

Organic–inorganic hybrids based on liquid crystalline symmetrical imine (8Z)-N-(4-((Z)-(4-pentylphenylimino)methyl)benzylidene)-4-pentylbenzenamine (AZJ1) with two aliphatic chains and TiO_2_ nanomaterials were obtained and investigated taking into account thr crystallographic form of titanium dioxide i.e., anatase versus rutile. The type of TiO_2_ influences the mesomorphic properties of imine AZJ1, as observed by differential scanning calorimetry (DSC) and polarizing optical microscopy (POM) techniques. Fourier-Transform Infrared Spectroscopy (FT-IR) was used to investigate the interactions of oxygen vacancies located on the TiO_2_ surface with the studied AZJ1 imine together with studying the influence of temperature. Both imine:TiO_2_ anatase versus rutile hybrids possessed the highest occupied molecular orbital (HOMO) levels of about −5.39 eV (AZJ1:anatase) and −5.33 eV (AZJ1:rutile) and the lowest unoccupied molecular orbital (LUMO) levels of about −2.24 eV. The presence of TiO_2_ in each hybrid did not strongly affect the redox properties of imine AZJ1. Organic devices with the configuration of ITO/TiO_2_/AZJ1 (or AZJ1:TiO_2_ anatase versus rutile)/Au were fabricated and investigated in the presence and absence of visible light irradiation with a light intensity of 93 mW/cm^2^. Finally, to analyze defects in the constructed organic devices we used thermal imaging and atomic force microscopy (AFM). The addition of TiO_2_ in both crystallographic forms has a positive influence on layer-forming properties that manifests itself as a very homogenous heat distribution for the whole sample.

## 1. Introduction

Imines (azomethines), the condensation product of diamine/amine and aldehyde/dialdehyde, are an interesting class of organic materials, especially due to its applicability in organic devices, with an inexpensive and short purification being required [1]. Imines/polyimines are mainly synthesized by a one- or two-step condensation reaction of adequate monomers in a solution with only water as a side-product of the reaction, and for this reason they are called ecologically-friendly compounds [2,3]. Proposed by scientists, simple chemical reactions of imines/polyimines for photovoltaics corresponds to the Green Chemistry rules [4]. Currently, a lot of new imines with various symmetries are synthesized to be used as thermal, biological or liquid crystalline materials [5,6,7,8]. Moreover, some imines are tested in organic devices, such as solar cells [9,10,11,12,13,14]. 

Furthermore, the presence of a free electron pair in the nitrogen atom of the imine bond allows acid–base interactions, and various organic and inorganic acids were used as Bronsted acids to doped imines [1]. However, the quest for novel compounds and hybrids is still an ongoing process in material science. In our previous works, we investigated hybrid materials based on TiO_2_ in anatase and imines with various structures and symmetries [15,16,17,18]. Our study showed that, depending on the chemical structure of imines, titanium dioxide in the anatase form changed selected properties of imines in more or less evident manner, probably due to the effect of different conformations of the investigated imines, geometrical symmetry and level of crystallinity. 

It is worth mentioning the interesting work published by Pola et al. [19], where the Ti^4+^ complexes obtained from Schiff base ligands reacted with donor atoms such as S and N under solvothermal conditions. Schiff base metal complexes can be applied to Green Chemistry, taking into consideration photosynthesis, and used in the mineralization of organic pollutants to less harmful byproducts and oxygen generation [19]. 

Our previous results inspired us to think about how liquid crystalline imine and their hybrids with TiO_2_ could be applied in organic devices. Numerous articles concerning the investigation of liquid crystalline imines with different shapes, symmetry and molecular weights can be found [7,8]. However, to best of our knowledge, this is the first paper to discuss how the crystallographic form of TiO_2_ influences the selected properties of liquid crystalline imines. Other scientists analysed the influence of TiO_2_ on selected properties of various polymers. For example, Jang et al. [20] presented enhanced diffraction efficiency in a photorefractive liquid crystal cell with poly(9-vinylcarbazole)-infiltrated mesoporous TiO_2_ layers, while Tercjak et al. [21] showed the arrangement of conductive TiO_2_ nanoparticles in hybrid inorganic/organic thermosetting materials using liquid crystals.

To analyse defects in, for example, solar cells, various techniques are used, however thermal imaging is a fast and simple method for locating defects [22,23,24,25]. In our work, a first time thermographic camera was used to detect the location of defects in the created devices and electrical behavior of imine and its hybrids with TiO_2_. 

It should be stressed that titanium dioxide is one of the richest compounds on our planet, and a very appealing material for a variety of applications [26,27,28,29,30,31]. In fact, TiO_2_ has been applied in: ultraviolet photodetectors, organic lights emitting diodes, organic solar cells, and photocatalytic water-splitting technology. Toward reducing the sensitivity of devices to oxygen and water vapor it is cheap and useful idea to incorporate TiO_2_ into the organic devices [32]. In organic solar cells, TiO_2_ could be applied as component of the (i) active layer; (ii) hole transporting layer; or (iii) interlayer.

The aim of this work was to create organic–inorganic hybrids based on (8Z)-N-(4-((Z)-(4-pentylphenylimino)methyl)benzylidene)-4-pentylbenzenamine (AZJ1) and titanium dioxide, and analyze the influence of TiO_2_ in anatase and rutile forms on the selected properties of imine, such as structural, thermal and electrochemical properties.

Organic–inorganic hybrids based on the liquid crystalline imine and TiO_2_ were obtained, and were investigated based on four main factors, as follows:

(i) influence of the TiO_2_ in the organic layer on liquid crystalline properties of imine AZJ1,
(ii) influence of the crystallographic form of TiO_2_ on HOMO-LUMO levels of AZJ1,
(iii) influence of the crystallographic form of TiO_2_ on IR properties of AZJ1,
(iv) influence of the crystallographic form of TiO_2_ on morphology properties of AZJ1 and created devices.

The investigations of the created inorganic–organic hybrids and structure–property relationship might form the basis for a “supramolecular engineering” approach for opto-electronic compounds. This comprises the purpose of their thermal, optical and redox properties.

## 2. Experimental

All chemicals and reagents were used as received from Sigma-Aldrich (Saint Louis, Missouri, USA). Details about synthesis of AZJ1 can be find in [33,34] and Supplementary Information (Figures S1–S3, Table S1). 

### 2.1. Preparation of AZJ1:TiO_2_ Mixture

To prepare mixture of azomethine and TiO_2_ a proper amount of azomethine, TiO_2_ (anatase or rutile) were mixed in chloroform to receive 5 mg/mL solution with ratio 3:2 azomethine:TiO_2_. The solution was dropped into microscope glass slide and heated up to c.a. 60 °C and kept in this temperature for c.a. 3 h to evaporate chloroform. For POM measurements, the glass slide was covered with another glass, and for DSC, the mixture was scratched with a spatula and put into aluminium pans. For ultraviolet–visible spectrophotometry (UV-Vis) measurements to 0.5 mg of AZJ1 1 mL of chloroform was added. To prepare samples of AZJ1:TiO_2_ (anatase or rutile) TiO_2_ was added in ratio azomethine:TiO_2_ equal to 3:2. Solution of pure TiO_2_ (anatase or rutile) had concentration c.a. 0.33 mg/mL.

### 2.2. Construction and Characterisation of Organic Devices

Samples for photocurrent measurements were prepared on ITO-patterned glass substrate (Osilla S211) to form a ITO/TiO_2_/AZJ1 (or AZJ1:TiO_2_)/Au multilayer structure. First, the ITO-coated glass substrate was cleaned by ultrasonication in acetone and isopropanol for 20 min and oxygen plasma for 30 s. TiO_2_ (3Dnano P25 average particle size 21 ± 5 nm) was mixed with ethanol for 4 h on a magnetic stirrer to form a homogeneous suspension. Ready suspension was spun cast (2000 rpm, 40 s) to form a uniform film and the TiO_2_ layer was annealed for 60 min at 650 °C. Then, AZJ1 or mixture of AZJ1 with TiO_2_ (3:2 w/w) in a chlorobenzene solution was spun cast on top of the TiO_2_ layer. TiO_2_ in rutile form was received from PlasmaChem, the medium size of the particles is 2 ± 1 nm. TiO_2_ powder in anatase form was prepared by the sol-gel method. The average grain size of obtained TiO_2_ (anatase) was found to be about 170 nm. 

Gold electrodes were deposited by thermal evaporation in a vacuum (5 × 10^−6^ mbar). The current–voltage (I–V) characteristics were measured under illumination of AM1.5 solar simulator (Oriel 150 W). Light power density was measured by Newport Oriel P/N 91150V reference solar cell.

**Figure 1:**
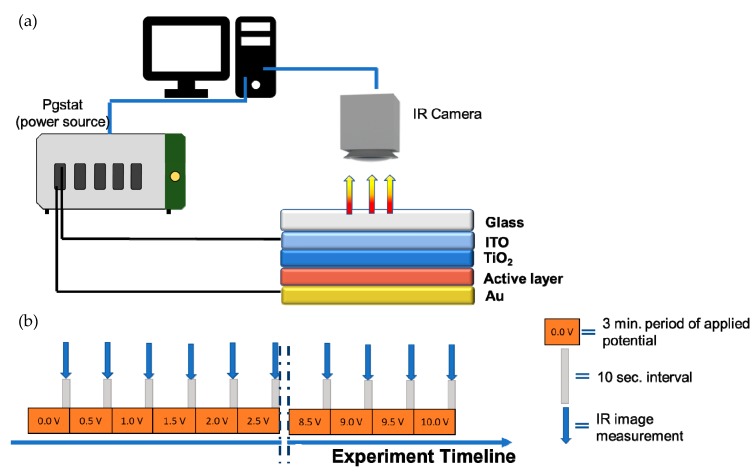
Thermography setup used to record images at different potentials (**a**); experimental timeline visualization presenting steps during thermographic experiment (**b**).

Thermal behavior was observed using a thermographic camera (VIGOcam v50, VIGO System S.A, Ożarów Mazowiecki, Poland) while applying bias voltage between 0 and 10 V and using a multichannel potentiostat-galvanostat (PGStat Autolab M101, Metrohm, Barendrecht, The Nederland) connected to computer (see Figure 1a). The experiment was design to apply voltage in programmed way (Figure 1b) as follows: the potential was applied in range from 0 V to 10 V with 0.5 V step between different values during three minutes for each voltage. The current response was recorded during three-minute intervals and each step was separated with a 10 second window, when the IR image was collected maintaining the value of the applied potential of the current step. The work of both the camera and power source was controlled via computer software.

### 2.3. Measurements

Temperature-dependent Fourier transform middle-infrared absorption measurement (FT-IR, Bruker, Ettlingen, Germany) was performed using a Bruker VERTEX 70v vacuum spectrometer equipped with an Advanced Research System liquid helium DE-202A cryostat and water-cooled helium compressor ARS-2HW working in a closed cycle manner. All spectra were obtained in the spectral range of 4000–400 cm^−1^ with a resolution of 2 cm^−1^ and 32 scans per each spectrum. The set temperature was measured with an accuracy of ±0.1 °C. The pure AZJ1 sample mixed with KBr and compressed into a pellet was measured first during heating and then subsequent cooling in the temperature range of 17–100 °C. Additionally, room-temperature FT-IR spectra of pre-prepared samples of two AZJ1–TiO_2_ mixtures (using anatase or rutile TiO_2_) were obtained with the same spectrometer and spectral parameters. All the spectra were recorded using OPUS 7.0 software and presented with Origin 2017 Pro.

The textures of imine and their mixture with TiO_2_ (anatase or rutile) were taken using Nikon Eclipse LV100 POL polarizing microscope (Tokyo, Japan) equipped with Fine Instruments WTMS-14C heating stage at cooling/heating rate 10 °C/min.

DSC measurements (Perkin Elmer, Waltham, Massachusetts, USA) were done using a Perkin Elmer DSC8000 calorimeter. The temperature was calibrated on the onsets of melting points of water and indium. The sample was hermetically sealed in aluminium pans of 30 l. Measurements were done during cooling/heating at a rate of 10 °C/min in a nitrogen atmosphere.

Samples for AFM measurements (Agilent Technologies Santa Clara, California, USA) were prepared by spin-coating (1000 rpm, 40 s). AFM imaging of sample topography was acquired at room temperature with the Agilent 5500 microscope working in non-contact mode. Setpoint and gains were adjusted to each measurement to obtain clear images without noise. Topography images were collected at several randomly-chosen areas.

Electrochemical measurements were carried out using a Metrohm Autolab PGSTAT M204 potentiostat (Barendrecht, The Nederland) and the electrochemical cell contained a glassy carbon electrode (diam. 2 mm), a platinum rod and Ag/AgCl as working, counter and reference electrodes, respectively. Potentials are referenced with respect to ferrocene (Fc), which was used as the internal standard. Cyclic voltammetry experiments were conducted in a standard one-compartment cell, in acetonitrile (≥99.9%, Honeywell), under argon. 0.2 M Bu_4_NPF_6_ (Alfa Aesar, 99%) was used as the supporting electrolyte. The concentration of compounds was equal to 1.0 × 10^−6^ mol dm^−3^. The deaeration of the solution was achieved by argon bubbling through the solution for about 15 min prior to the measurement. All electrochemical experiments were carried out at ambient temperature and pressure. 

The transmission UV-Vis spectra were acquired using laboratory build system based on a computer-controlled monochromator (Cornerstone 260 1/4m, Newport Corp., Irvine, CA, USA) equipped with two holographic gratings and automatic filter sorter. Light from the monochromator passing through a quartz cuvette was measured using amplified Si-photodiode (Newport) connected to lock-in amplifier (SR-530, Stanford Research System), optical chopper placed between light source and the monochromator. 

## 3. Results and Discussion 

### 3.1. Temperature-Dependent FT-IR Spectrum of AZJ1

**Figure 2:**
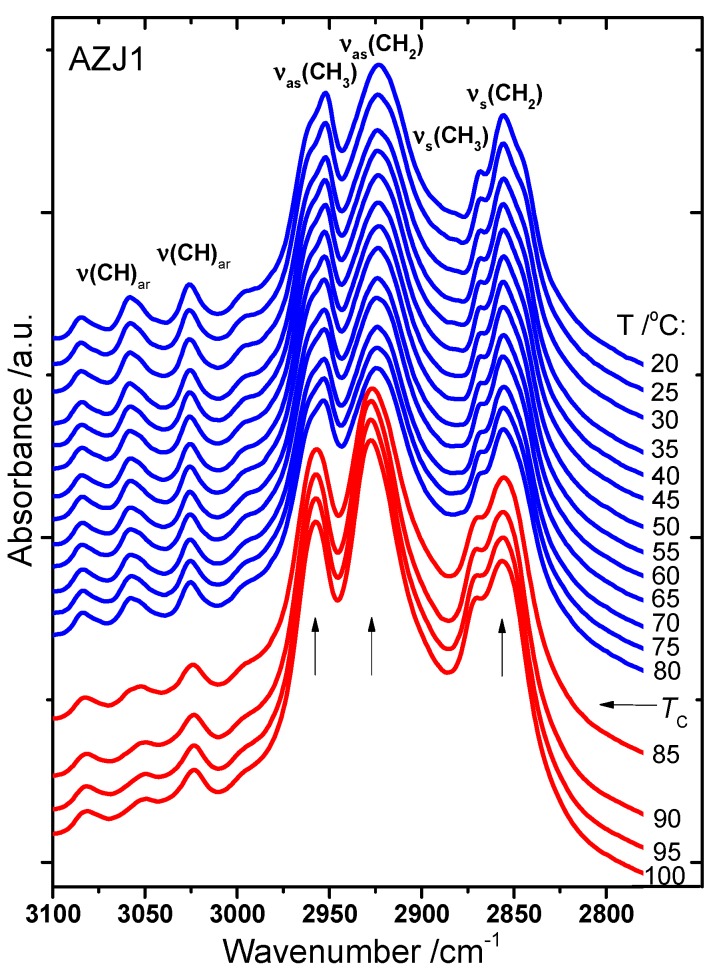
Temperature evolution of the FT-IR spectrum of AZJ1 obtained during heating in the spectral range of 3100–2780 cm^−1^. Ordered and liquid crystal phases are marked blue and red, respectively. The most changed bands upon the transition are marked with vertical arrows.

**Figure 3:**
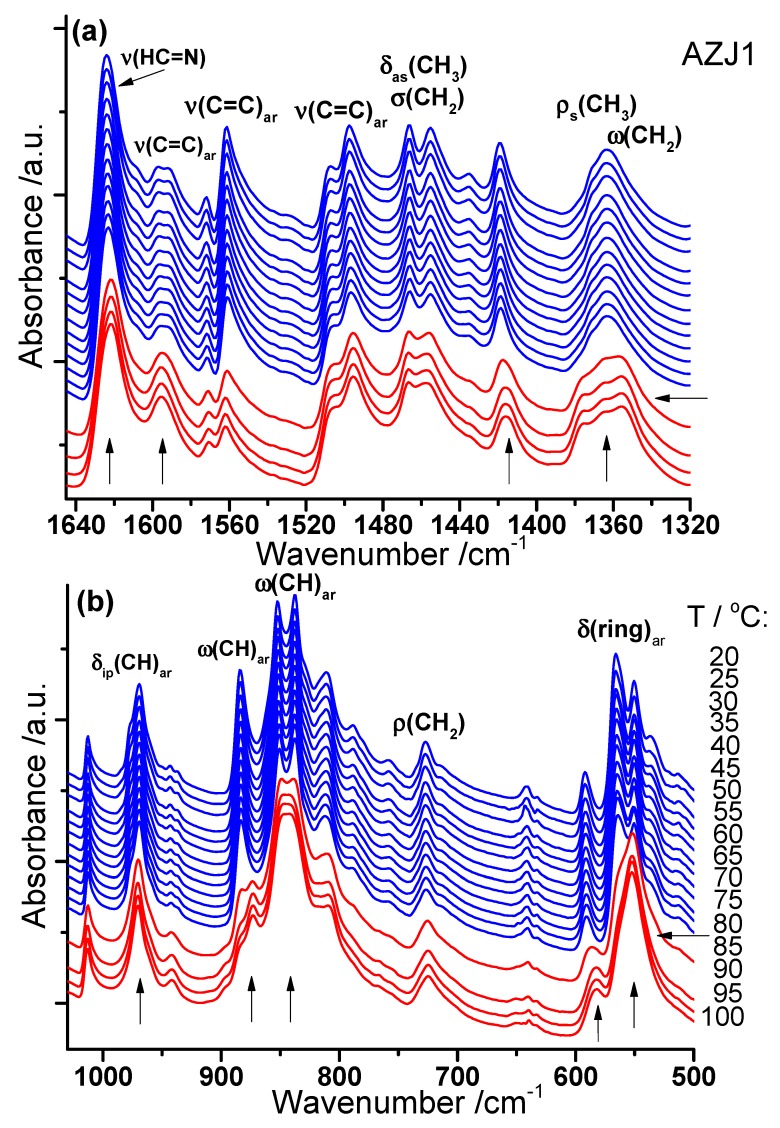
Temperature evolution of the FT-IR spectrum of AZJ1 obtained during heating in two spectral ranges: (**a**) 1645–1320 cm^−1^ and (**b**) 1030–500 cm^−1^. Ordered and liquid crystal phases are marked blue and red, respectively. The most changed bands upon the transition are marked with vertical arrows.

Temperature-dependent FT-IR spectroscopy was applied in order to obtain more information about the nature of the phase transition between the crystalline and liquid crystalline (LC) phases. The spectra obtained in the ordered phase and LC phase are marked with a blue and red colour, respectively (Figure 2 and Figure 3). Additionally, the horizontal and vertical arrows point to the phase transition region and the IR bands which changed the most during the transition. As the highest possible temperature in this experiment is 130 °C, we could not study the other transitions observed at much higher temperatures. Figure 3 presents temperature evolution of the IR spectrum of AZJ1 registered during heating in the high-wavenumber region (>2750 cm^−1^), where the ν(CH) stretching vibrations of aliphatic chains as well as aromatic rings occur. The bands connected to ν_as_(CH_2_) and ν_s_(CH_2_) vibrations of aliphatic chains usually appear at 2915 and 2850 cm^−1^, respectively, which corresponds to highly-ordered hydrocarbon chains with an all-trans conformation. In the case of AZJ1, asymmetric CH_2_ vibration appears at 2924 cm^−1^ at room temperature, thus at a relatively higher energy, indicating the existence of trans and cis isomers in the hydrocarbon chains. On conversion from the ordered to LC phase, some changes in this region of the IR spectrum are also clearly visible: the position of the band mentioned earlier shifts toward higher wavenumbers by about 3 cm^−1^. The bands connected to ν_as_(CH_3_) and ν_s_(CH_2_) modes, which are split into two components at room temperature, become single at the LC phase. 

Figure 3a shows the temperature evolution of the IR spectrum in the region where the internal vibrations within aliphatic chains, aromatic rings and imine groups occur. The transition from the crystalline (spectra marked with blue) to LC phase (spectra marked with red) is clearly visible. Splitting of the band connected to the aliphatic chains (ω(CH_2_) mode at 1363 cm^−1^ into two components: 1365 and 1357 cm^−1^) and shifting of the δ(C_ar_CH) mode at 1419 cm^−1^ toward lower wavenumbers can be observed above the transition to the LC phase. In turn, the bands connected to ν(C=C)ar modes at 1597 and 1592 cm^−1^ become single and the band connected to ν(HC=N) mode shifts slightly toward lower wavenumbers above the transition to the LC phase.

Some significant changes are also visible in the lower wavenumber ranges below 1030 cm^−1^ (see Figure 3b) during transition to the LC phase. Namely, three double bands, connected most probably to the bending vibrations of aromatic rings, δ(CH)_ar_ (at 977 and 969 cm^−1^), ω(CCH)_ar_ (at 852 and 838 cm^−1^), and δ(ring)_ar_ (at 565 and 550 cm^−1^) become single. In turn, the sharp and intensive band associated to ω(CCH)ar mode at 884 cm^−1^ splits into two components. Also, the other band ascribed to the δ(ring)_ar_ or δ(CNC) mode at 592 cm^−1^ shifts distinctively toward lower wavenumbers by about 9 cm^−1^ and broadens on conversion to the LC phase.

The spectroscopic results show that practically all molecular groups of which the AZJ1 compound is composed—p-substituted benzene rings, hydrocarbon segments and imine groups—are involved in the ordered crystal–liquid crystal transition, which occurs at 85 °C, as observed using the IR method. On subsequent cooling of the AZJ1 sample down from 100 °C to 17 °C, the reverse transition from the LC to crystalline phase was observed at 47 °C (thermal hysteresis in the transition temperature: ~40 °C), which is in a good agreement with the DSC results.

### 3.2. FT-IR Spectra of AZJ1 and Its Mixtures with TiO_2_

**Figure 4:**
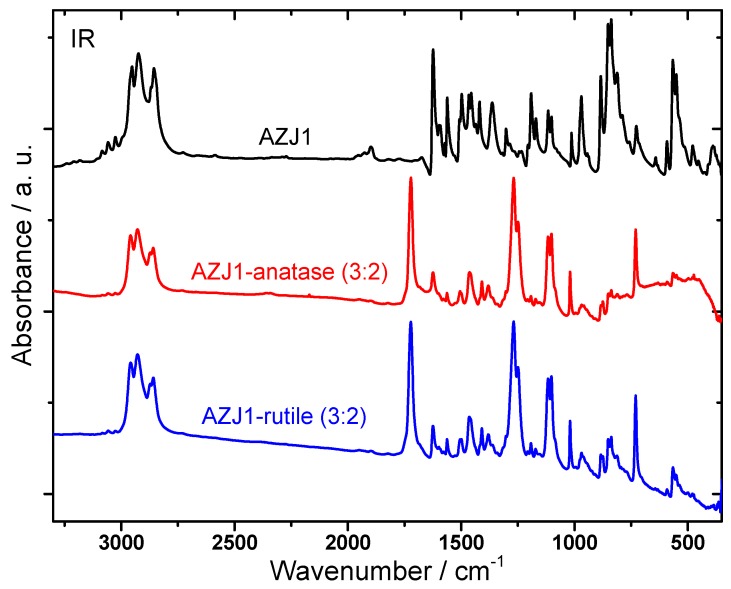
Comparison of room-temperature FT-IR spectra of pure AZJ1, AZJ1–TiO_2_ (anatase) and AZJ1–TiO_2_ (rutile) mixtures marked with different colors.

Figure 4 presents the FT-IR spectra of AZJ1 and two mixtures: AZJ1:TiO_2_ (anatase) and AZJ1:TiO_2_ (rutile) obtained at room temperature. The spectra of both AZJ1:TiO_2_ mixtures are very similar to each other, considering the band positions and intensities, and are at the same time different from the spectrum of pure AZJ1. This suggests that anatase as well as rutile TiO_2_ used for mixtures’ preparation affects the interactions between TiO_2_ and AZJ1 in the same way.

Most of the bands observed in the IR spectrum of AZJ1 are also visible in the spectra of AZJ1:TiO_2_ mixtures, but their intensities are much lower. In the high wavenumber range between 3000 and 2750 cm^−1^ the bands connected to the stretching modes of methyl and methylene groups within aliphatic chains are of comparable intensities in AZJ1 and its mixtures. In turn, the ν(CH)_ar_ stretching modes in aromatic rings are present in the mixtures but are characterized by much lower intensities. In the lower wavenumber range the most interesting bands which are present only in the spectra of mixtures are those of the highest intensities: the band at 1722 cm^−1^ probably associated with imine bonds in AZJ1 shifts to higher wavenumbers in the mixture with TiO_2_. Our spectroscopic results indicate strong bonding interactions occurring between AZJ1 and oxygen vacancies placed on the TiO_2_ surface. Interestingly, these interactions are of the same type in case of both minerals, anatase and rutile, used in the preparation of mixtures.

### 3.3. POM and DSC Study

**Figure 5:**
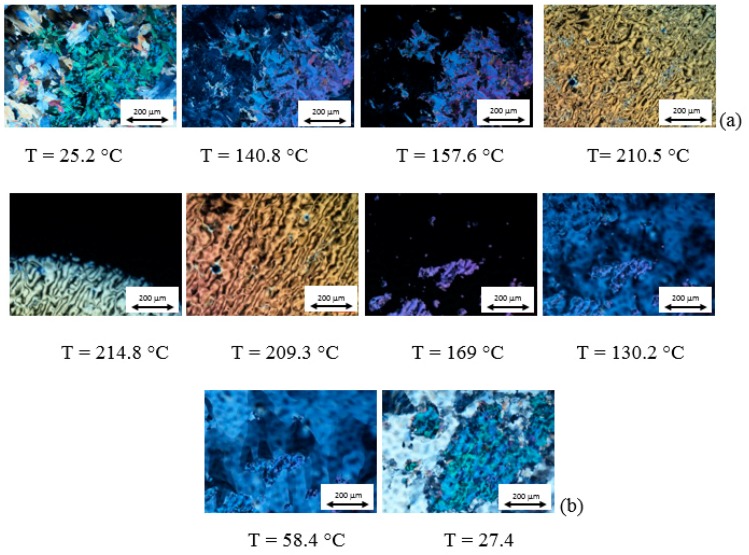
Photomicrographs of the optical textures registered at various temperatures for the AZJ1 during heating (**a**) and cooling (**b**) at a rate of 10 °C/min.

Optical textures and DSC curves of AZJ1 are shown in Figure 5 and Figure S4, respectively. Taking into account these results and those obtained by FTIR, it is seen that AZJ1 exhibits three different liquid crystalline phases during heating, and four during cooling. Moreover, the phase transition temperature to isotropic liquid is very high, circa 230 °C. 

**Figure 6:**
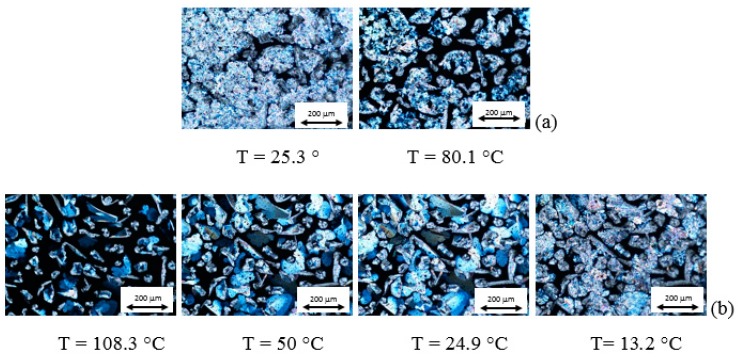
Photomicrographs of the optical textures registered at various temperatures for the AZJ1:TiO_2_ (anatase) during heating (**a**) and cooling (**b**) at rate 10 °C/min.

Optical textures and DSC curves registered for mixture AZJ1:TiO_2_ (anatase) are presented in Figure 6 and Figure S5a, respectively. There is a significant difference between pure azomethine and its mixture with TiO_2_. Firstly, the changes in textures are well visible. Besides, there is only one liquid crystalline phase during heating and three during cooling for the mixture with anatase. Moreover, temperatures of the phase transitions are shifted towards lower temperatures (see Table 1). 

**Figure 7:**
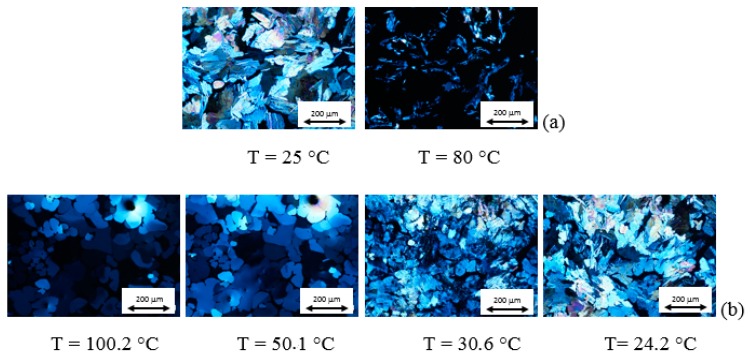
Photomicrographs of the optical textures registered at various temperatures for the AZJ1:TiO_2_ (rutile) during heating (**a**) and cooling (**b**) at 10 °C/min.

POM and DSC results registered for the mixture AZJ1:TiO_2_ (rutile), shown in Figure 7 and Figure S5b, are different from that for pure azomethine and the mixture with TiO_2_ (anatase). The mixture AZJ1:TiO_2_ (rutile) exhibits one liquid crystalline phase during heating and three during cooling similarly to AZJ1:TiO_2_ (anatase). The phase transition temperatures for both mixtures are also similar. In DSC measurements for AZJ1:TiO_2_ (rutile) a phase transition between liquid crystal and isotropic liquid is not visible, both for heating and cooling. This might be due to a smaller mass of the sample. It is shown that the structure of TiO_2_—anatase or rutile—affects the properties of the azomethine AZJ1. Temperatures and enthalpy changes of the phase transitions for all three samples obtained from DSC are gathered in Table 1. 

### 3.4. AFM Study

The topographies of thin layers of pure imine AZJ1 and its mixtures with TiO_2_ are different, as shown in Figure S6. The large domains chaotically arranged on the surface are visible for the thin films of pure imine AZJ1. A few high domains are visible on the surface of TiO_2_ (anatase) mixed with AZJ1, while pure anatase forms sharp threadlike aggregates. Chaotically arranged protrusions are noticeable on the surface of films containing TiO_2_ (rutile). The number of features visible in Figure S6d,e are similar, but the protrusions in the pure TiO_2_ (rutile) layer look much sharper than in the mixture with AZJ1. The value of roughness (Rms) is different in each case. For pure AZJ1, roughness is the lowest (26.73 nm) and for the mixture AZJ1:TiO_2_ (anatase), it is the highest (39.17 nm).

### 3.5. UV and Electrochemical Study

In order to evaluate the electrochemical properties of AZJ1 and its mixture with two different crystalline forms of TiO_2_ (rutile and anatase), cyclic voltammetry was used. Generally, a small difference in Eg and HOMO-LUMO levels of pure AZJ and its mixtures with both anatase and rutile (ratio 3:2 w/w) form of TiO_2_, about 0.06 eV and 0.11 eV, respectively, were observed (Table 2). 

**Figure 8:**
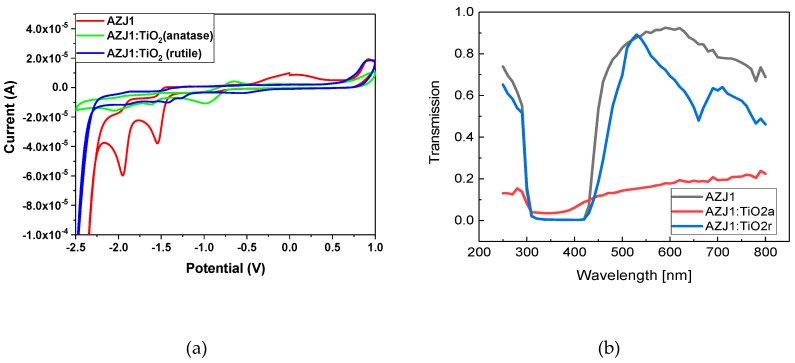
(**a**) Cyclic voltammograms (CV) of the mixture of AZJ1 with two different TiO_2_ crystallographic structures—rutile and anatase—in solution. CV sweep rate ν = 100 mV s^−1^, 0.2 M Bu4NPF6 in acetonitrile. (**b**) UV-Vis transmission spectra (transmission spectra of TiO_2_ suspensions in chloroform are presented in Figure S7).

The addition of TiO_2_ to imines affects both the value and shape of the peaks, as it can be seen in Figure 8a.

For AZJ1, apart from the offset redox processes another partially-reversible oxidation–reduction reaction occurs in the range of −2.25 V to −1.40 V, with reductions maxima at −1.95 V and −1.55V, and their corresponding oxidations maxima at -1.85 V and −1.44 V. Furthermore, an additional oxidation signal can be observed at 0.03 V as a broad prick. In the mixture, the presence of TiO_2_ in both forms affects the intermediate oxidation–reduction processes without major influence on the terminal ones, causing shifts and decreases in the intensity of the signals. In the case of anatase, the maxima of reduction at −2.05 V, −1.63 V −0.98 V and oxidation at −1.92 V, −1.55 V and −0.65 V demonstrate the first two picks decrease in intensity and shift to the lower values of potentials with what is consistent with the shift of whole spectra in the same direction. Moreover, an additional reversible redox process can be observed in the potential of −1 V to −0.5 V. Regarding the AZJ1 with rutile mixture, distinguishable reduction maxima at −1.80 V, −1.41 V, −1.24 V and −0.57 V, and oxidation maxima at −1.88 V, −1.36 V, −1.27 V and −0.48 V can be observed. The influence of TiO_2_ in the rutile form on imine has smaller influence on the potential at which the processes occur, however, it significantly decreases their intensity.

The transmittance spectra of AZJ1 in solution reveals just one broad intensive absorption band at 280–460 nm and two other maxima at 700 nm and 781 nm. The presence of TiO_2_ in the anatase form mixed with AZJ1 causes a scattering effect, which results in a decrease in the intensity of the spectra. Due to the flattening and widening of the band, the determination of the maximum of the band was not possible. On the other hand, the presence of the same amount of the rutile form of TiO_2_ in the other sample results in absorption band separation, unveiling the presence of two partially-overlapped bands with maxima at 660 nm and 782 nm. Those results suggest titanium dioxide in the rutile form has bigger effect on the electron transfer in the azomethine molecule.

### 3.6. Organic Devices with AZJ1 and AZJ1:TiO_2_

**Figure 9:**
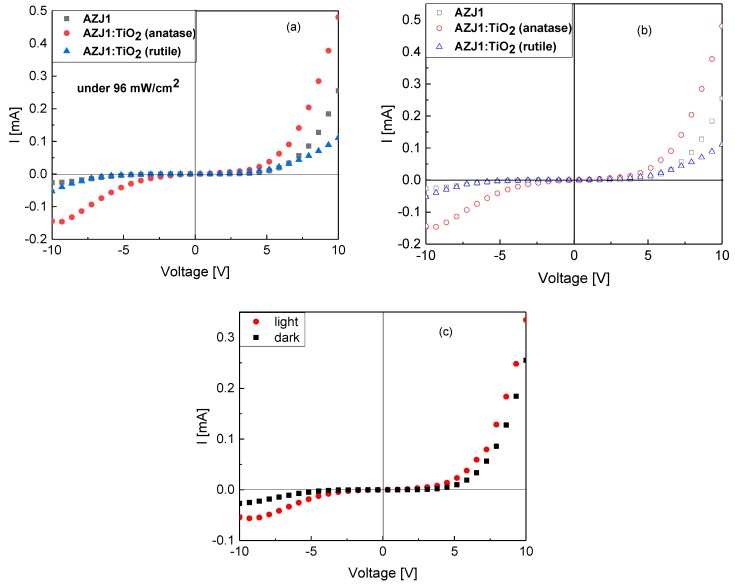
I–V curves of the ITO/TiO_2_/organic layer/Au under 96 mW/cm^2^ illumination (**a**) and in the dark (**b**); of the ITO/TiO_2_/AZJ1/Au in the dark and under illumination (**c**).

The I–V characteristic for devices with an architecture of ITO/TiO_2_/organic layer/Au was measured in the dark and under solar illumination. For azomethine and its mixtures with TiO_2_ (anatase and rutile), the generation of photocurrent does not occur, as there is no difference between the dark and light I–V characteristic. Figure 9 shows the comparison between these three devices under illumination and in the dark together with I–V characteristics for ITO/TiO_2_/AZJ1/Au in the dark and under illumination, as an example. The highest intensity of current was observed for AZJ1:TiO_2_ (anatase) and the lowest was AZJ1:TiO_2_ (rutile). 

Figure 9b shows the current-voltage characteristics of ITO/TiO_2_/organic layer/Au devices in the dark. The forward voltage corresponding to the Au electrode was negative for all investigated devices. The imine/Au or imine:TiO_2_/Au interface probably forms a blocking or Schottky barrier. On the other hand, the TiO_2_/imine, the TiO_2_/imine:TiO_2_(anatase), or the TiO_2_/imine:TiO_2_(rutile) layer interface is probably an ohmic contact. A similar effect was observed by Sharma et al. [35] for poly (phenyl azomethine furane) thin films devices in the dark, as well as in our previous studies [17,18]. 

**Figure 10:**
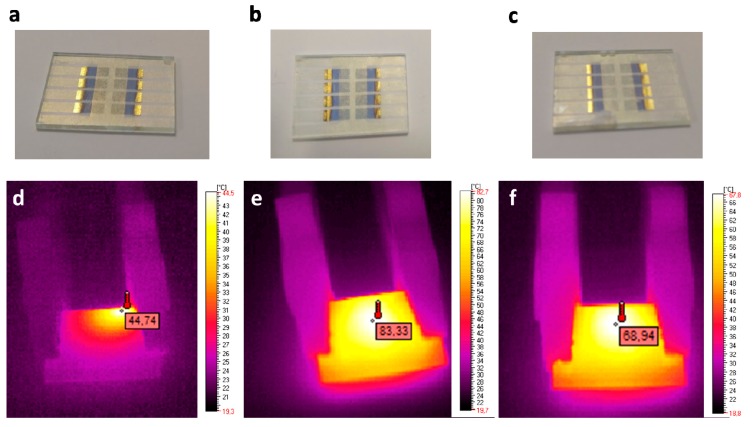
Images of the prepared devices: glass/ITO/TiO_2_/AZJ1/Au (**a**); glass/ITO/TiO_2_/AZJ1:TiO_2_(rutile)/Au (**b**); glass/ITO/TiO_2_/AZJ1:TiO_2_(anatase)/Au (**c**) and IR images of those devices respectively containing only AZJ1 (**d**), and mixture of AZJ1 with TiO_2_ in the form of rutile (**e**) and anatase (**f**) at an applied potential of 8.5 V.

Three devices with an architecture of glass/ITO/active layer/Au, where the active layer was AZJ1, AZJ1:TiO_2_(rutile) and AZJ1:TiO_2_(anatase) (Figure 10), were studied using a thermographic camera, applying the potential to observe possible defects of the constructed device. The experimental setup was designed in a specific way to detect any possible fluctuation of current which could explain the thermal responses of the samples. 

**Figure 11:**
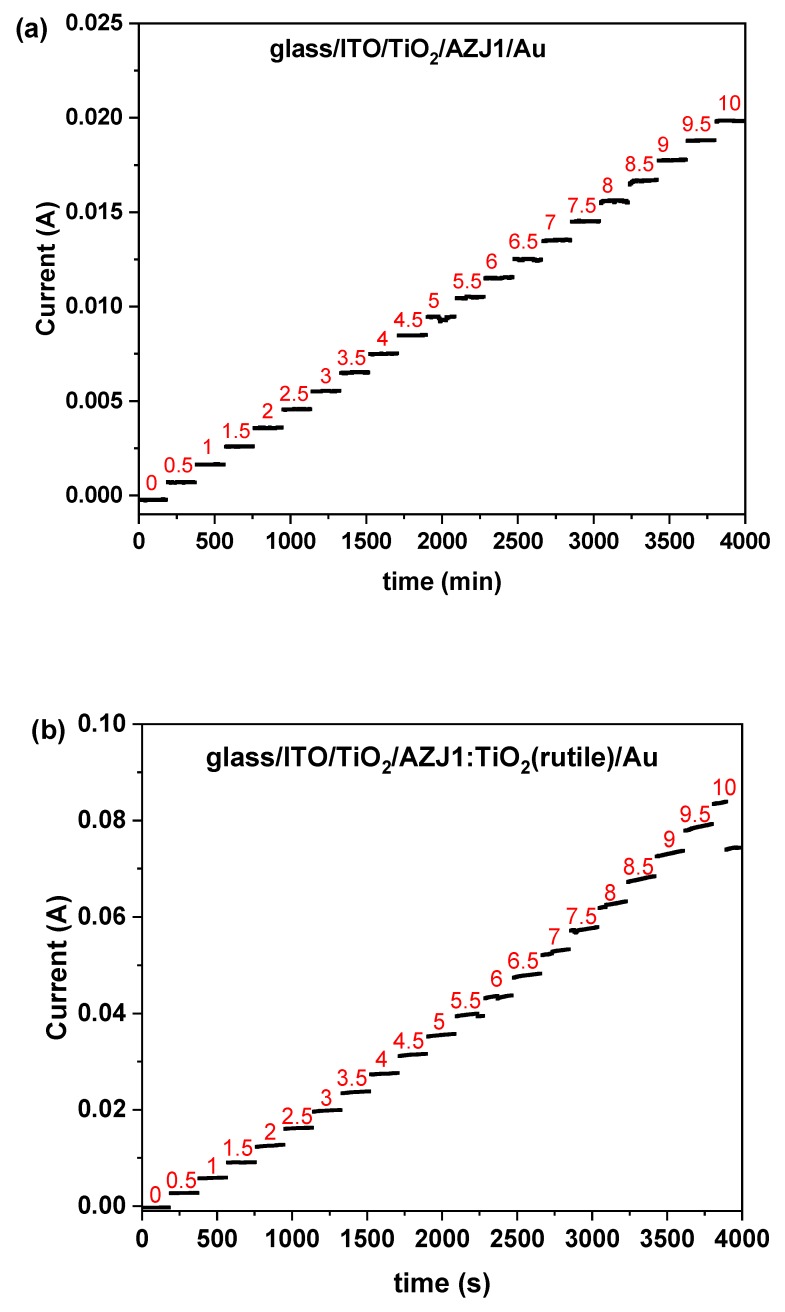

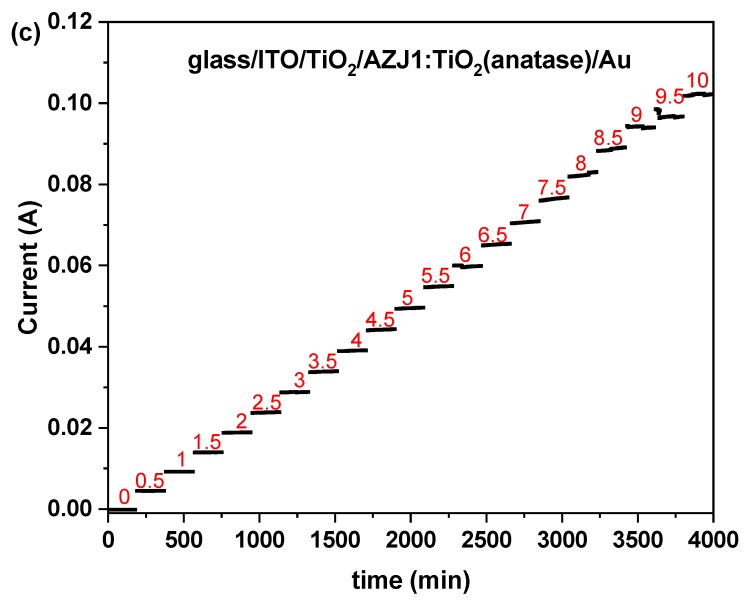
The correlation of current flow versus time at different applied potentials for constructed devices containing only AZJ1 (**a**), and mixture of AZJ1 with TiO_2_ in form of rutile (**b**) and anatase (**c**), where numbers from 0 to 10 are the applied voltage in [V].

The correlation of time-dependent current flow at different applied potentials can be seen in Figure 11. Based on the observed tendency, all samples showed conductive behavior as expected, with differences in obtained values of resistance. In principle, resistance decreases linearly with increasingly applied voltage, which is also related to increments of temperature, as expected for conducting layers. Resistance ranged from 727.2 Ω, 183.6 Ω and 112.9 Ω to 504.0 Ω, 134.4 Ω and 97.8 Ω respectively for samples containing AZJ1, AZJ1:TiO_2_(rutile) and AZJ1:TiO_2_(anatase). Those findings are consistent with the results of electrical properties obtained for devices during illumination, where it was observed that the lowest resistance was for glass/ITO/TiO_2_/AZJ1:TiO_2_(anatase)/Au and the highest was for glass/ITO/TiO_2_/AZJ1/Au. Only in the case of devices with the architecture of glass/ITO/TiO_2_/AZJ1:TiO_2_(rutile)/Au, a small decline in current was observed when 10 V was applied on bias. This can be related to only one melting transition observed in DSC for the mixture AZJ1:TiO_2_(rutile) at 68.85 °C, since 10 V applied on a sample caused an increase of temperature to 70.4 °C, which is in the range of above-mentioned transition. For other samples this phenomenon was not observed, because pure AZJ1 and its mixture with the anatase form of titanium dioxide has other transitions at higher temperatures, which explains the higher thermal resistance of layers. 

**Figure 12:**
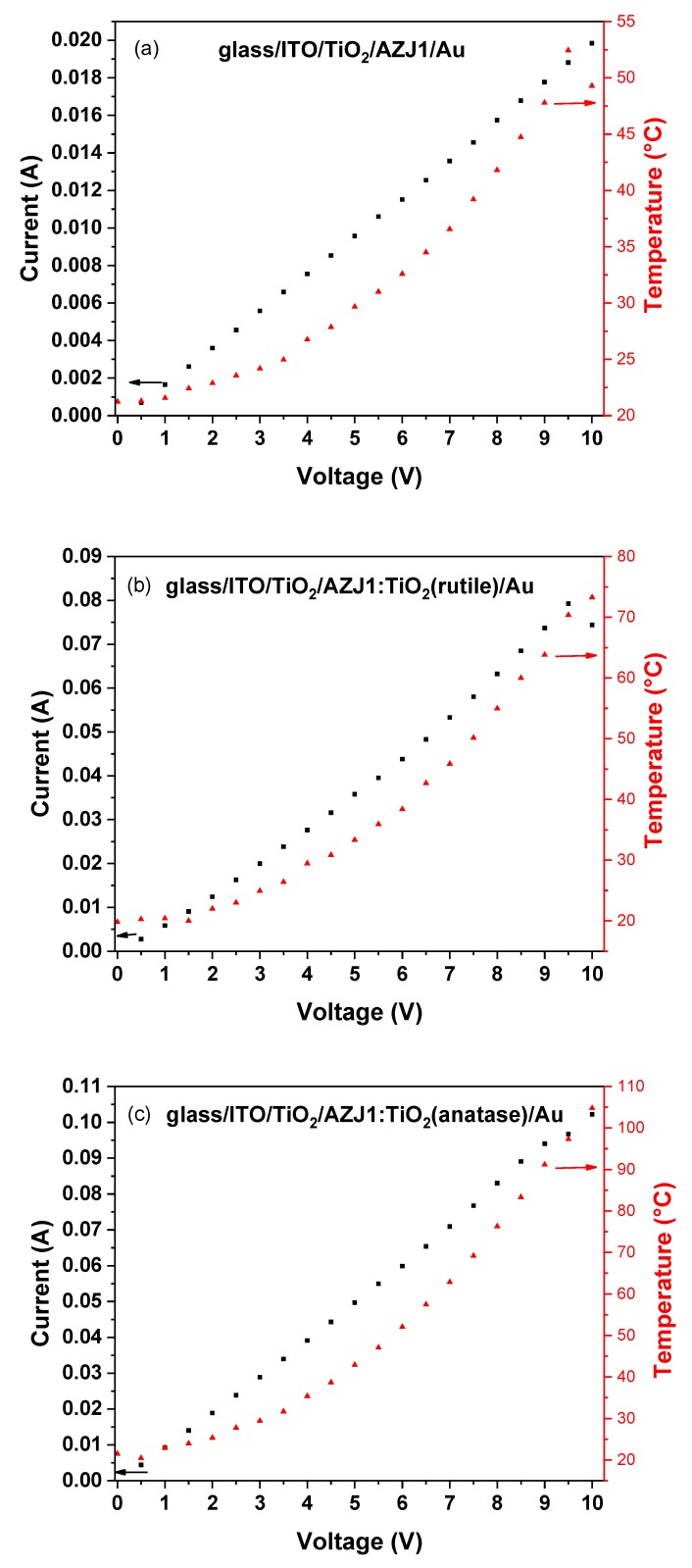
The correlation of current flow and temperature versus applied potential for constructed devices containing only AZJ1 (**a**), and mixture of AZJ1 with TiO_2_ in the form of rutile (**b**) and anatase (**c**).

For better visualization of thermal and electrical responses at different applied potentials, their plots were prepared for studied samples (Figure 12). For all samples, increasing the potential induced linear responses of the samples, as mentioned previously. However, the thermal response to current flow displayed a logarithmic-like shape of the curves. Since the potential was applied for three minutes and during this time the current was maintained in the same range for the whole step, any effects due to heating can be disregarded. It was observed that the temperature remained stable, even after the three minute period. The logarithmic-like shape becomes a plain at voltages above 9 V. For most samples, this is also related with thermally-induced transitions in the layers containing azomethine. The most visible transition can be observed for devices containing pure AZJ1 (Figure 12a): for this material in DSC at 70.28 °C, an endothermic transition was observed with the highest enthalpy change for all observed samples. This transition resulted in a decrease of temperature as a possible consequence of energy absorption required for structural transition. Despite the temperature change, the electrical response was not changed, since for this sample other transitions at higher temperatures were observed. This might suggest that only the aliphatic chains gained some mobility without affecting the general structure of the molecule. Obtained studies confirm the influence of the crystalline structure of inorganic titanium dioxide addition on the behavior of formed layers, and the absence of transition at temperatures above 140 °C is possibly related to the presence of higher-ordered structures.

Analyzing the images considering the thermal distribution on the sample surface, as seen in Figure 10, the content of the active layer has a significant influence on the temperature distribution of a sample’s surface. For the device containing pure AZJ1, the distribution of the temperature is mainly concentrated at one edge; the shortest way between external connections. This behavior can be explained by an inhomogeneous layer, where the thermal response highlights the conduction path. In this case, defective construction of the device partially explains the highest values of resistance and as a consequence, the lowest electrical parameter during illumination. The inhomogeneity can be influence by the molecular structure of imine, which tends to form uneven layers. On the other hand, the addition of TiO_2_ in both crystallographic forms has a positive influence on layer-forming properties, which manifests itself as a very homogenous heat distribution of the whole sample. 

## 4. Conclusions 

In summary, organic–inorganic hybrids based on imine and TiO_2_ together with organic devices based on these compounds were obtained and characterized in detail. In the constructed devices, TiO_2_ was applied as both a hole transporting material and as a component of the active layers. Our study shows that the addition of TiO_2_ in both crystallographic forms has a positive influence on layer-forming properties, which manifests itself as a very homogenous heat distribution across the whole sample, as confirmed by thermographic camera. Those findings are consistent with the result of electrical properties obtained for devices during illumination, where it was observed that the lowest resistance was for glass/ITO/TiO_2_/AZJ1:TiO_2_(anatase)/Au and the highest was for glass/ITO/TiO_2_/AZJ1/Au.

Moreover, the crystallographic form of TiO_2_ decreases the number of mesophases from three in AZJ1 to one for the AZJ1:TiO_2_ (anatase) hybrid, to only the melting point being observed for the AZJ1:TiO_2_ (rutile) hybrid. Finally, our FT-IR results indicate strong bonding interactions occurred between AZJ1 and oxygen vacancies placed on the TiO_2_ surface independently from the crystallographic form of TiO_2_. 

## Figures and Tables

**Table 1:** Transition temperatures and associated enthalpy changes of AZJ1 and AZJ1:TiO_2_ upon heating and cooling at a rate of 10 °C/min.

Code	Transition Temperatures (°C) (ΔH, J/g)	
	Heating	Cooling
**AZJ1 (m = 4.06 mg)**	70.28 (36.967), 147.52 (8.576), 210.96 (1.610), 233.05 (2.409)	233.59 (−3.200), 212.27 (−1.415), 148.57 (−8.122), 62.03 (−2.627), 49.67 (−27.171)
**AZJ1:TiO_2_ (m = 2.75 mg) anatase**	68.79 (11.146), 143.65 (1.431)	143.34 (−1.340), 58.90 (−0.707), 49.85 (−0.386), 46.89 (−8.174)
**AZJ1:TiO_2_ (m = 2.04 mg) rutile**	68.85 (12.384)	59.46 (−0.205) 48.07 (−0.070) 44.09 (−1.085)

**Table 2:** Electrochemical and energy level parameters of AZJ1 mixed with TiO_2_.

Sample Code	E_ox_^onset^ [V]	E_red_^offset^ [V]	E_HOMO_ [eV]	E_LUMO_ [eV]	E_g_ [eV]
**AZJ1**	0.9	−2.26	−5.35	−2.31	3.04
**AZJ1:TiO_2_, anatase (3:2)**	0.94	−2.33	−5.39	−2.24	3.15
**AZJ1:TiO_2_, rutile (3:2)**	0.88	−2.34	−5.33	−2.23	3.10

## Supplementary Materials

The following are available online at https://www.mdpi.com/1996-1944/12/7/1097/s1, Figure S1: Scheme of AZJ1 synthesis, Figure S2: ^1^H NMR of AZJ1 in CDCl_3_, Figure S3: FT-IR spectrum of AZJ1 obtained at room temperature with marked positions of the most prominent bands, Table S1: List of band positions (cm^-1^) of the FT-IR spectrum of AZJ1 with tentative assignments based on literature [1,2,3]. Figure S4: DSC curves of the imine AZJ1 registered during heating and cooling cycle at rate 10 °C/min, Figure S5: DSC curves of the imine AZJ1:TiO_2_ (anatase) (a) and AZJ1:TiO_2_ (rutile) (b) registered during heating and cooling cycle at rate 10 °C/min, Figure S6: AFM topography images of the imine and its mixtures with TiO_2_ with mass ratio 3:2: (a) AZJ1 molecule layer, (b) AZJ1:TiO_2_ anatase mixture layer, (c) TiO_2_ anatase layer, (d)AZJ1:TiO_2_ rutile mixture layer, (e) TiO_2_ rutile layer, Figure S7: Transmission spectra of TiO_2_ nanocrystals suspensions in chloroform, lower transmittance of anatase as compared to rutile for higher wavelengths results from higher scattering on larger particles.
