# Supplementary material for: Influence of TiO2 Nanoparticles on Liquid Crystalline, Structural and Electrochemical Properties of (8Z)-N-(4-((Z)-(4-pentylphenylimino)methyl)benzylidene)-4-pentylbenzenamine
Source: Materials (Basel). 2019 Apr 2;12(7):1097. doi: 10.3390/ma12071097 (PMC6480637; doi:10.3390/ma12071097)
Supplement: Supplementary file 1 [file materials-12-01097-s001.pdf]

*materials*

*Supplementary Materials*

# Influence of $TiO_2$ nanoparticles on liquid crystalline, structural and electrochemical properties of (8Z)-N-(4-((Z)-(4-pentylphenylimino)methyl)benzylidene)-4-pentylbenzenamine

**Anna Różycka [1], Krzysztof Artur Bogdanowicz [2], Natalia Górska [3], Jakub Rysz [1], Monika Marzec [1,*], Agnieszka Iwan [4,*], Robert Pich [4], Adam Januszko [4]**

[1] Institute of Physics, Jagiellonian University, S. Lojasiewicza 11, 30-348 Krakow, Poland; a.rozycka@doctoral.uj.edu.pl (A.R.); jakub.rysz@uj.edu.pl (J.R.)
[2] Military Institute of Engineer Technology, Obornicka 136, 50-961 Wroclaw, Poland; bogdanowicz@witi.wroc.pl (K.A.B.)
[3] Faculty of Chemistry, Jagiellonian University, Gronostajowa 2, 30-387 Kraków, Poland; gorska@chemia.uj.edu.pl
[4] General Tadeusz Kosciuszko Military University of Land Forces, Wroclaw; MULF Wroclaw, Faculty of Security Studies, Czajkowskiego 109, 51-147 Wroclaw, Poland; robert.pich@awl.edu.pl (R.P.); adam.januszko@awl.edu.pl (A.J.)
* Correspondence: monika.marzec@uj.edu.pl (M.M.); agnieszka.iwan@awl.edu.pl (A.I.)

Received: 11 March 2019; Accepted: 29 March 2019; Published: date

**Synthesis of AZJ1**

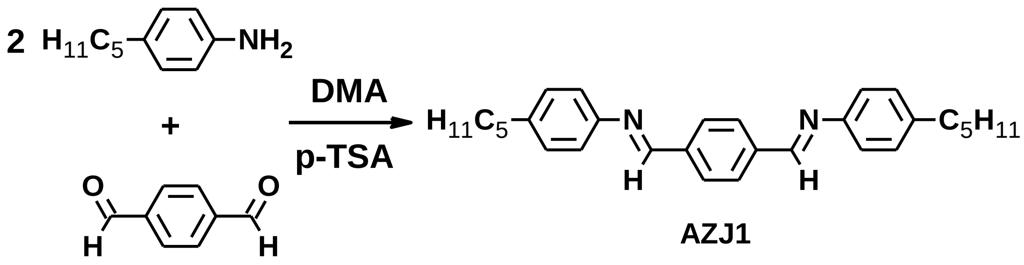


**Figure 1.** Scheme of AZJ1 synthesis.

Sample was characterized with $^1H$ NMR, using deuterated chloroform ($CDCl_3$) as a solvent with a Jeol ECZ-400 S spectrometer ($^1H$ - 400 MHz) with delay time 5 s. Measurements were carried out at room temperature on 10-15% (w/v) sample solutions.

$^1H$ NMR (400 MHz, $CDCl_3$), δ [ppm]: 8.52 (s, 2H, -HC=N-), 7.99 (s, 4H), 7.23-7.17 (m, 8H), 2.62 (t, 4H), 1.61 (m, 4H), 1.35- 1.30 (m, 8H), 0.89 (t, 6H).

$^{13}C$ NMR (75 MHz, $CDCl_3$, TMS) [ppm]: δ= 158.55 (HC=N), 149.24, 141.46, 138.61, 129.98, 128.96, 120.87, 35.46, 31.46, 31.18, 22.53, 14.03.

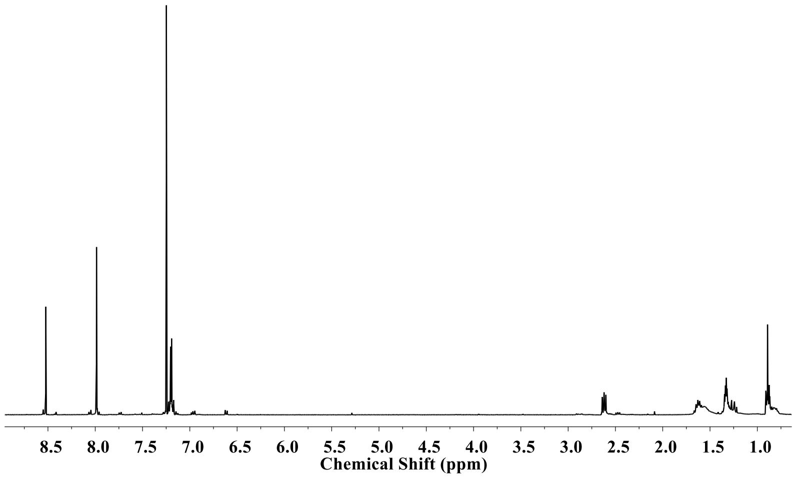


**Figure 2.** $^1$H NMR of AZJ1 in $CDCl_3$.

## Spectroscopic characterization of AZJ1 at room temperature (FT-IR)

The investigated AZJ1 compound was preliminary studied by middle infrared absorption spectroscopy at room temperature in order to confirm its proper composition and molecular structure. The IR spectrum and tentative description of the observed bands are presented in Figure S3 and Table S1 in Supplemental Material. The spectrum consists mainly of bands connected to stretching and bending vibrations of aliphatic chains, aromatic rings and the –HC=N– imine groups located between the aromatic rings. The most intensive bands at 852 and 838 $cm^{-1}$ are connected to the $\omega(CCH)_{ar}$ out of plane bending vibrations of 1,4-substituted benzene rings. Considering stretching vibrations within aromatic rings, several sharp bands of medium intensity between 1600–1490 $cm^{-1}$ ascribed to $\nu(C=C)_{ar}$ vibrations and several bands of low intensity between 3110–3000 $cm^{-1}$ ascribed to $\nu(CH)_{ar}$ vibrations are present. The characteristic strong band connected to the $\nu(HC=N)$ stretching mode of the imine groups is located at 1624 $cm^{-1}$.

As the AZJ1 compound contains two terminal aliphatic chains, strong bands in the spectral range of 3000–2750 $cm^{-1}$ connected to the $\nu(CH_2)$ and $\nu(CH_3)$ symmetric and asymmetric stretching vibrations can also be observed. In turn, the bands connected to $-CH_2$ and $-CH_3$ bending vibrations are visible in the range of 1470–1300 $cm^{-1}$ in the IR spectrum. The characteristic rocking vibration $\varrho(CH_2)$, which appears when long aliphatic chains are present, is split into two components what suggests existing of closely packed methylene chains. In addition to fundamental vibrations, overtones and combination tones are also present as low intensity bands located in three spectral ranges: 3280–3140 $cm^{-1}$, 2750–2500 $cm^{-1}$, and 2000–1640 $cm^{-1}$. Summing up, all functional groups expected in the IR spectrum of AZJ1 could be observed what confirms proper composition of the investigated compound.

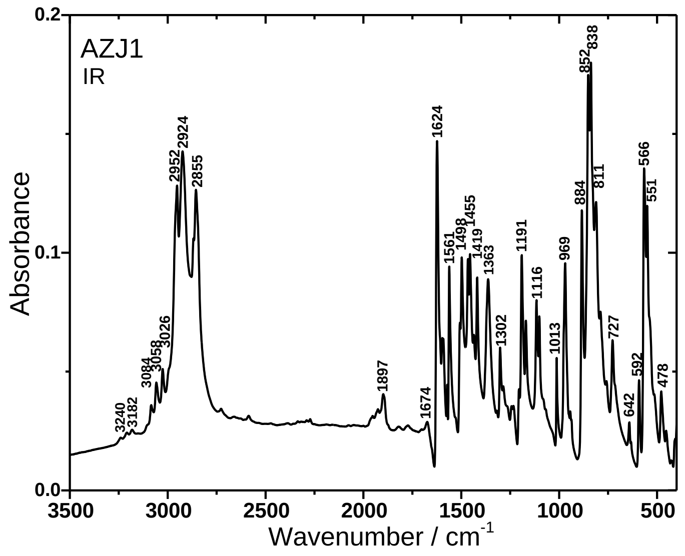


**Figure 3.** FT-IR spectrum of AZJ1 obtained at room temperature with marked positions of the most prominent bands.

**Table 1.** List of band positions ($cm^{-1}$) of the FT-IR spectrum of AZJ1 with tentative assignments based on literature [1–3].

| Band position | Tentative assignments | Band position | Tentative assignments |
|---|---|---|---|
| 3240 w | $2\nu(HC{=}N)$ | 1244 w | - |
| 3208 w | $2\nu(C{=}C)_{ar}$ | 1233 w | - |
| 3182 w | $2\nu(C{=}C)_{ar}$ | - | - |
| 3106 w | $\nu(CH)_{ar}$ | 1191 m | $\delta(CH)_{ar}$ ip |
| 3084 w | $\nu(CH)_{ar}$ | 1170 m | $\delta(CH)_{ar}$ ip |
| 3058 w | $\nu(CH)_{ar}$ | 1116 m | $\delta(CH)_{ar}$ ip or $\nu(CC)_{aliph}$ |
| 3026 w | $\nu(CH)_{ar}$ | 1101 m | $\delta(CH)_{ar}$ ip or $\nu(CC)_{aliph}$ |
| 2993 sh | - | 1013 m | - |
| 2960 sh | $\nu_{as}(CH_3)$ | 977 sh | $\delta(CH)_{ar}$ ip |
| 2952 s | $\nu_{as}(CH_3)$ | 969 m | $\delta(CH)_{ar}$ ip |
| 2924 vs | $\nu_{as}(CH_2)$ | 884 s | $\omega(CCH)_{ar}$ |
| 2868 sh | $\nu_s(CH_3)$ | 852 vs | $\omega(CCH)_{ar}$ |
| 2855 s | $\nu_s(CH_2)$ | 838 vs | $\omega(CCH)_{ar}$ |
| 2844 sh | $\nu_s(CH_2)$ | 811 s | $\omega(CCH)_{ar}$ |
| 1953 vw | $2\tau(CH_3)$ | 789 m | - |
| 1926 vw | $2\tau(CH_3)$ | 758 sh | - |
| 1899 w | - | - | - |
| 1674 w | $2\omega(CCH)_{ar}$ $\nu(HC{=}N)$ | 727 m | $\rho(CH_2)_{long\ chain}$ |
| 1624 vst | | 714 sh | $\rho(CH_2)_{long\ chain}$ |
| 1597 m | $\nu(C{=}C)_{ar}$ | 642 w | - |
| 1592 m | $\nu(C{=}C)_{ar}$ | 633 sh | - |
| 1572 w | $\nu(C{=}C)_{ar}$ | - | - |
| 1561 m | $\nu(C{=}C)_{ar}$ | 592 m | $\delta(ring)_{ar}$ oop or $\delta(CNC)$ |
| 1523 vw | $\nu(C{=}C)_{ar}$ | 566 s | $\delta(ring)_{ar}$ oop or $\delta(CNC)$ |
| 1507 m | $\nu(C{=}C)_{ar}$ | 551 s | $\delta(ring)_{ar}$ oop or $\delta(CNC)$ |
| 1498 m | $\nu(C{=}C)_{ar}$ | 538 sh | - |
| 1466 m | $\delta_{as}(CH_3)$ | 478 w | - |
| 1455 m | $\sigma(CH_2)$ | 453 vw | - |
| 1436 sh | - | 389 w | - |
| 1419 m | $\delta(C_{ar}CH)$ | | |
| 1371 m, sh | $\rho_s(CH_3)$ | | |
| 1363 m | $\omega(CH_2)$ | | |
| 1302 m | $\nu(C_{ar}–N)$ | | |
| 1285 w | $\nu(C_{ar}–N)$ | | |

(w – weak; sh – shoulder; m – medium; s – strong; vs – very strong; ν – stretching; δ – bending; in plane (ip) modes: ρ – rocking, σ – scissoring; out of plane (oop) modes: ω – wagging, τ – twisting; ar – aromatic; aliph – aliphatic).

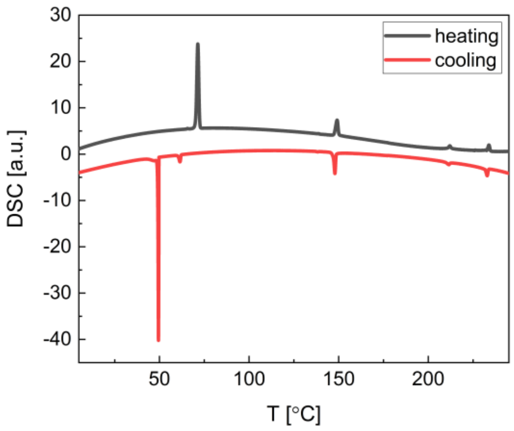


**Figure 4.** DSC curves of the imine AZJ1 registered during heating and cooling cycle at rate 10 °C/min.

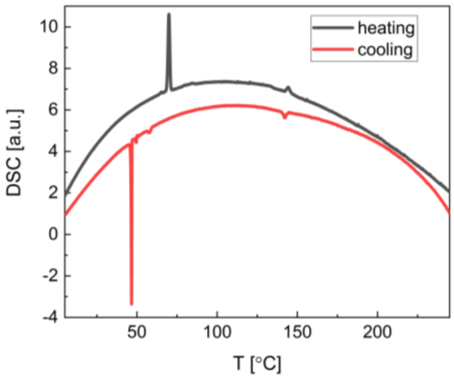


(a)

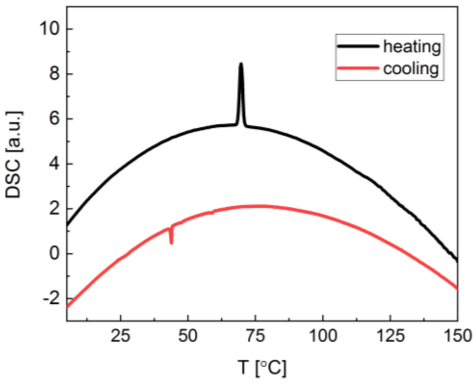


(b)

**Figure 5.** DSC curves of the imine AZJ1:$TiO_2$ (anatase) (a) and AZJ1:$TiO_2$ (rutile) (b) registered during heating and cooling cycle at rate 10 °C/min.

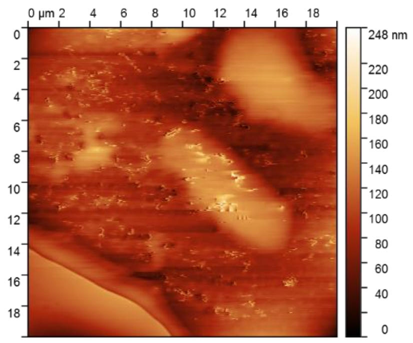


a) AZJ1 RMS 26.73 nm

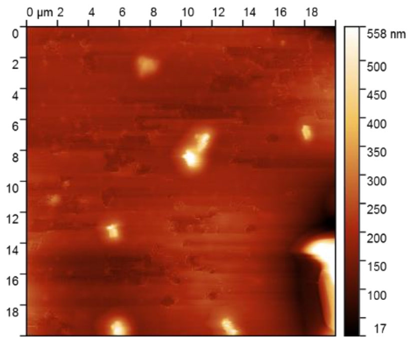


b) AZJ1:$TiO_2$ (anatase) RMS 59.66 nm

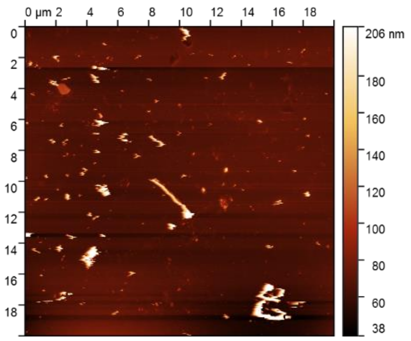


c) $TiO_2$ (anatase) RMS 19.51 nm

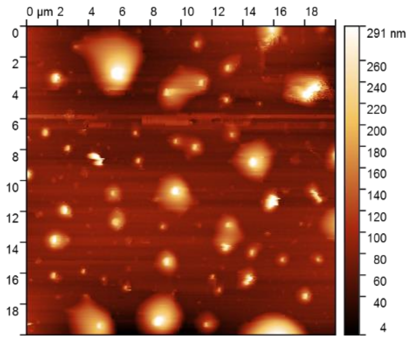


d) AZJ1:$TiO_2$ (rutile) RMS 39.17 nm

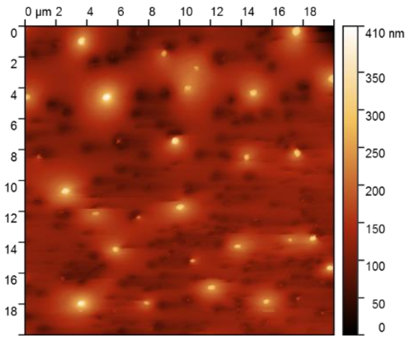


e) $TiO_2$ (rutile) RMS 33.32 nm

**Figure 6.** AFM topography images of the imine and its mixtures with $TiO_2$ with mass ratio 3:2: (a) AZJ1 molecule layer, (b) AZJ1:$TiO_2$ anatase mixture layer, (c) $TiO_2$ anatase layer, (d) AZJ1:$TiO_2$ rutile mixture layer, (e) $TiO_2$ rutile layer.

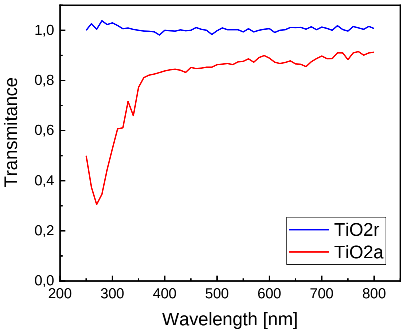


**Figure S7.** Transmission spectra of $TiO_2$ nanocrystals suspensions in chloroform, lower transmittance of anatase as compared to rutile for higher wavelengths results from higher scattering on larger particles.

1. Cozan, V.; Avadanei, M.; Perju, E.; Timpu, D. *Phase Transitions* **2009**, *82*, 607–619.
2. Lebovka, N.; Dadakova, T.; Lysetskiy, L.; Melezhyk, O.; Puchkovska, G.; Gavrilko, T.; Baran, J.; Drozd, M. *J. Mol. Struct.* **2008**, *887*, 135–143.
3. Różycka, A.; Iwan, A.; Filapek, M.; Górska, N.; Bogdanowicz, K.A.; Dabczynski, P.; Rysza, J.; Pociecha, D.; Hreniak, A.; Marzec, M. *Liq. Cryst.* **2018**, *45*, 831–843.
